# Deciphering Hybrid Larch Reaction Norms Using Random Regression

**DOI:** 10.1534/g3.118.200697

**Published:** 2018-10-17

**Authors:** Alexandre Marchal, Carl D. Schlichting, Rémy Gobin, Philippe Balandier, Frédéric Millier, Facundo Muñoz, Luc E. Pâques, Leopoldo Sánchez

**Affiliations:** * INRA, Research Unit on Forest Breeding, Genetics and Physiology (UR 0588 AGPF), Centre de recherche Val-de-Loire, 45075 Ardon, France; † Department of Ecology & Evolutionary Biology, University of Connecticut, Storrs, CT 01230; ‡ IRSTEA, Research Unit on Forest Ecosystems (EFNO), Domaine des Barres, F-45290 Nogent-sur-Vernisson, France

**Keywords:** phenotypic plasticity heterosis tree rings traits soil water availability multi-trait model

## Abstract

The link between phenotypic plasticity and heterosis is a broad fundamental question, with stakes in breeding. We report a case-study evaluating temporal series of wood ring traits of hybrid larch (*Larix decidua* × *L. kaempferi* and reciprocal) in relation to soil water availability. Growth rings record the tree plastic responses to past environmental conditions, and we used random regressions to estimate the reaction norms of ring width and wood density with respect to water availability. We investigated the role of phenotypic plasticity on the construction of hybrid larch heterosis and on the expression of its quantitative genetic parameters. The data came from an intra-/interspecific diallel mating design between both parental species. Progenies were grown in two environmentally contrasted sites, in France. Ring width plasticity with respect to water availability was confirmed, as all three taxa produced narrower rings under the lowest water availability. Hybrid larch appeared to be the most plastic taxon as its superiority over its parental species increased with increasing water availability. Despite the low heritabilities of the investigated traits, we found that the expression of a reliable negative correlation between them was conditional to the water availability environment. Finally, by means of a complementary simulation, we demonstrated that random regression can be applied to model the reaction norms of non-repeated records of phenotypic plasticity bound by a family structure. Random regression is a powerful tool for the modeling of reaction norms in various contexts, especially perennial species.

The link between phenotypic plasticity and heterosis has been well documented for annual plants, notably maize (Janick 1999; Gallais 2009). Usually, hybrids are able to stay productive in environments where one or both parents’ performances drop. This enhanced stability is referred to as ’hybrid homeostasis’, and can be responsible for the environmental conditionality of the expression of heterosis (Knight 1973). Comparing larch growth in several contrasted sites, we recently highlighted homeostasis for hybrid larch (HL, *Larix decidua* Mill. × *L. kaempferi* (Lamb.) Carr., and the reciprocal cross), a highly productive conifer cultivated for wood in Western Europe and North America (Marchal *et al.* 2017). Because the plasticity of some traits can be a tool for the homeostasis of the whole organism, phenotypic plasticity is suspected to play a key role in the construction of hybrid larch heterosis.

Phenotypic plasticity, in its narrow-sense definition, is the ability of a genotype to produce several phenotypes depending on the environmental conditions. It can be studied by means of reaction norms (Schlichting and Pigliucci 1998). A reaction norm is an equation, or simply a graphical representation, of the value taken by a phenotypic trait along an environmental gradient. Estimating reaction norms is not a trivial task. In particular, exposing the same genotype to different environments may be experimentally challenging, depending on the biological model. In some cases, the only solution is to expose related individuals to the different environments, relying on their genetic relationship to draw the common, additively inherited, component of the reaction norms (*e.g.*, Gibert *et al.* 2004; Valladares *et al.* 2006). The random regression model is an extension to the classical quantitative genetics model (Kirkpatrick and Heckman 1989), and as such it can predict the additive component of reaction norms, and give access to causal components of population variation. In addition, Murren *et al.* (2014) demonstrated with a large meta-analysis that when comparing reaction norms of close species or populations, changes in shapes (*i.e.*, slope, curvature) were generally higher than the changes in the intercepts (*i.e.*, the taxon means), suggesting the need of high-order modeling. In that view, random regression modeling has also the valuable capability to fit complex curves for reaction norms.

Random regression is a special case of covariance function (Meyer 1998). Covariance functions present a particular interest in quantitative genetics, as they allow the representation of quantitative genetics parameters as functions. For instance, the cattle breeding literature is rich in illustrations of heritabilities and genetic correlations estimated as functions of time in the milk production context (*e.g.*, Miglior *et al.* 2007; Muir *et al.* 2007; Jamrozik *et al.* 2010). Although covariance functions and random regression have been often suggested for the modeling of reaction norms (Kirkpatrick and Heckman 1989; De Jong and Bijma 2002; Schaeffer 2004), their application is still rare for most taxa (Morrissey and Liefting 2016). For instance, in the forestry context, random regression has been used to model growth over time (Apiolaza and Garrick 2001; Wang *et al.* 2009), but not tree growth reaction norms over environmental gradients until very recently (Carnwath and Nelson 2016; Marcatti *et al.* 2017). Li *et al.* (2017) advocated for the use of random regression in forest trees, but the growth reaction norms they reviewed were only performed at the population scale, with fixed effects, without covariance functions. Nevertheless, because they are sessile, long-living, and because they record radial growth increments in the form of annual rings, trees are remarkable biological models for the study of phenotypic plasticity. The use of random regression has been suggested for the study of tree rings plasticity (Sánchez *et al.* 2013).

For a given tree, the succession of wood annual rings constitutes an archive of its plastic responses to the succession of past climatic environments. Indeed, trees growth is conditioned by solar radiation, temperature and precipitation. They are particularly sensitive to drought, as this can eventually lead to lethal embolism of their hydraulic system. As a response to water stress, trees have a range of plastic responses including modification of the cambial activity to control the sap flow rate and xylem resistance to embolism (Bréda *et al.* 2006; Rennenberg *et al.* 2006). Under a temperate climate, water availability is usually high in spring with cool temperatures and lower in summer with higher temperatures. Trees respond to this seasonal succession by producing an early wood, made of large cells with thin walls, progressively followed by a late wood, made of narrow cells with thick walls, allowing a rapid change in the trunk of the water conductance along the season (Sánchez-Vargas *et al.* 2007; Martinez-Meier *et al.* 2009; Bryukhanova and Fonti 2013). This succession of early and late wood is recorded in the wood as an annual growth ring.

In summary, trees present the remarkable feature of recording long repeated series of phenotypes that are known to respond plastically to the climatic environment, and differentially among individuals. This provides a gold mine of information for the study of phenotypic plasticity. First, based on a random regression approach, we leveraged this information to address the question of the role of phenotypic plasticity in the construction of HL heterosis for radial growth. Indeed, integrative heterosis as observed for HL stem circumference necessarily arises from the cumulation of heterosis occurring at the annual ring scale: this might be expressed differentially with respect to parental genotypes depending on water availability. Second, we described how the water availability affected the quantitative genetic parameters of HL and of its parental species. Finally, since most biological models do not archive repeated series of phenotypic plasticity records, we addressed the question of the generalizability of the method. Using simulations, we evaluated the robustness of the random regression approach for the modeling of reaction norms in the case where no repeated series of phenotypes per individual are available.

## Materials and Methods

The data are part of a multi-site progeny trial established in early 1997 on two environmentally contrasted sites at Saint-Appolinaire (SA, 45${}^{\circ }$58’N 4${}^{\circ }$26’E, 784 m a.s.l.) and Saint-Saud (SS, 45${}^{\circ }$31’N 0${}^{\circ }$48’E, 307 m a.s.l.) in central France. The site of SA is a relatively high-elevation site, on a steep slope with a southern aspect formerly planted with Douglas-fir; whereas the site of SS is a low elevation site on a former meadow, under oceanic influences.

Progenies were produced by control-crossing in the frame of a diallel mating design between 9 European larch (*Larix decidua*, EL) parents and 9 Japanese larch (*L. kaempferi*, JL) parents, producing pure species and HL full-sib progenies. A total of 327 EL, 472 JL and 1199 HL genetically distinct trees were ultimately available for analysis in SA; and 311 EL, 706 JL and 1261 HL in SS. The mating design is detailed in Supplementary 1, Tables S1 and S2, with the number of genotypes available for each combination of parents. The set-up is also described further in Marchal *et al.* (2017).

### Phenotypic data: wood formation records

**Figure 1:**
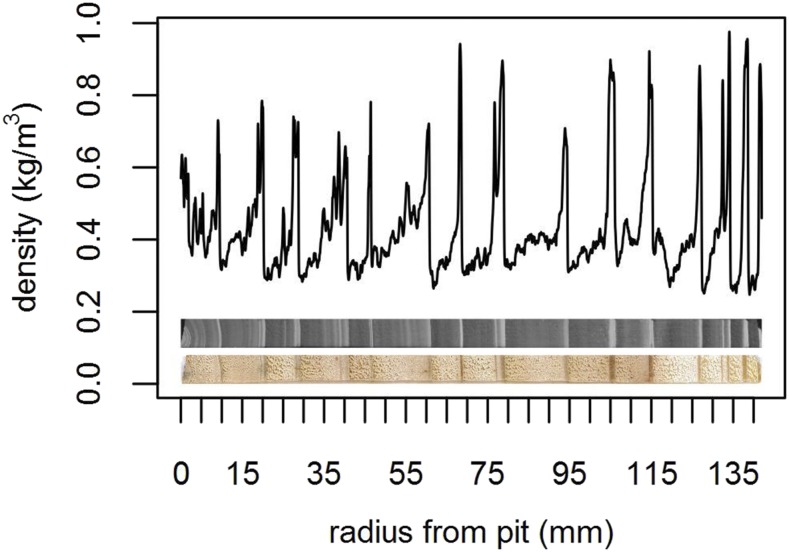
Microdensitometric profile from one wood core sample in Saint-Appolinaire. Bottom: wood core sample (pith on the left). Middle: X-ray radiography of the sample. Top: wood density variation (kg/m${}^{3}$) along the core (mm). The peaks correspond to late wood and delimit the annual growth rings.

One breast-height diameter increment core was collected from each tree from each site. For each diameter increment core, only one radius (the one exhibiting the fewest defects) was kept for further analysis. These radial increment cores were sawed in 2 mm thick boards and X-rayed to obtain microdensitometric profiles (Figure 1). From the alternating of early wood and late wood, the year of formation for each ring was identified (Regent Instruments Canada Inc. 2008). Ring width (RW) and ring mean density (RMD) were measured from the microdensitometric profiles. A total of 1998 increment cores in SA and 2278 increment cores in SS were collected. In SS, the increment cores were collected at three different periods: before the first thinning in 2003 from trees to be felled, and two later ones before and after the second thinning, that is in 2009 and in 2011. In SA, the collection was done in 2013. The average number of rings available per core was 13.2 in SA and 9.7 in SS (Supplementary 1, Fig. S1).

### Environmental data: soil water availability

The soil daily relative extractable water (REW) content was estimated using a water balance model. The REW varies between 1 (field capacity) and 0 (permanent wilting point, meaning the water is no longer available for the plants). We used an adapted, simplified implementation of Granier *et al.* (1999)’s daily water balance model. This implementation allowed two important features. First, it accounted for the temporal evolution of the stand. Indeed, during the growth of the trees, the canopy leaf area increases and subsequently increases the transpiration, whereas understorey shrubs and grass vegetation decrease. Second, the model could be run at the individual tree level. Indeed, the intra-site spatial heterogeneity of the soil storage capacity causes variation in the water availability at the individual tree level. Thus, the daily REW was estimated for each combination of individual tree within each site and year for which ring data were available. The water balance model is described in Supplementary 2.

To summarize a year of fluctuation of REW, we selected the lowest decile of the whole series of the year’s daily REW values (D1rew). This index was considered because of its easy interpretation (the REW below which lay the ∼35 driest days of the year), because of its likelihood to carry the information of drought events and therefore to affect the growth, and finally because it ensures a proper coverage of the environmental gradient (Supplementary 1, Fig. S2), unlike *e.g.*, indexes based on a drought threshold that may not be reached some humid years. The environmental gradient coverage guarantees the stability of the regression parameters and the quality of the subsequent analyses.

The index D1rew, derived from a complex water balance model, was compared to a simpler index MJJA defined as the sum of rain from May to August (inspired from Fallour-Rubio *et al.* (2009)), from which was subtracted the daily potential evapotranspiration (Supplementary 1, Fig. S2). Moreover, Bryukhanova and Fonti (2013) showed that for European larch, several traits including RW were more strongly correlated to the soil water deficit of year t−1 than to the deficit of the current year *t*. Therefore, we modeled the reaction norms along D1rew for year *t*, for year t−1, and MJJA for year *t* as three separate environmental gradients, and we compared the coefficient of determination ($R^{2}$) (Nakagawa and Schielzeth 2013; Johnson 2014) of the resulting reaction norms.

### Modeling of reaction norms by random regression model

Reaction norms were modeled using orthogonal Legendre polynomials (Kirkpatrick *et al.* 1990; Schaeffer 2004). Let $L_{m}\left(x^{\prime }\right)$ be the $m^{\text{th}}$ order Legendre polynomial of $x^{\prime },$ with $x^{\prime }$ the standardization of *x* on [-1, 1], that is $x^{\prime }=2\left[x-\text{min}\left(x\right)\right]/\left[\text{max}\left(x\right)-\text{min}\left(x\right)\right]-1.$ We fitted the following model for each taxon (EL, HL or JL):

$$\begin{array}{l} y_{ijkl}\left(x\right)=\sum_{m=0}^{M_{S}}s_{im}L_{m}\left(x^{\prime }\right)+\sum_{m=0}^{M_{A}}a_{jm}L_{m}\left(x^{\prime }\right)\\ +\sum_{m=0}^{M_{P}}p_{km}L_{m}\left(x^{\prime }\right)+r_{ijkl} \end{array}$$

where $y_{ijkl}\left(x\right)$ was the $l^{\text{th}}$ observation of individual *k*, of genotype *j*, from site *i*, with environment *x*. The site’s effect *s* was fixed. The additive effects *a* were random, and depended on the species. For EL and JL pure species, respectively, $a_{E}∼N\left(0,\Sigma_{AE}⊗A_{E}\right)$ and $a_{J}∼N\left(0,\Sigma_{AJ}⊗A_{J}\right)$, where ⊗ indicates a Kronecker product, A were the additive relationship matrices computed from the pedigree, and $\Sigma_{A}$ were the estimated variance-covariance matrices for additive effects. For the hybrid, $a_{H}=g_{E}+g_{J}$ with *g* the additive contributions from each parental species. Thus, $g_{E}∼N\left(0,\Sigma_{HE}⊗\frac{1}{2}A_{HE}\right)$ on the EL side and $g_{J}∼N\left(0,\Sigma_{HJ}⊗\frac{1}{2}A_{HJ}\right)$ on the JL side, with $\Sigma_{H}$ the estimated variance-covariance matrices for additive effects in hybridization (Stuber and Cockerham 1966). Given that the parents were supposed outbred and unrelated, the resulting relationship matrices $A_{HE}$ and $A_{HJ}$ reduced to identity matrices. The permanent environment, *i.e.*, the similarity from non-additive genetic origin between the repeated measures of the same individual tree (Mrode and Thompson 2005), was $p∼N\left(0,\Sigma_{P}⊗I_{P}\right)$. The residual was unstructured $r∼N\left(0,\sigma_{R}^{2}I_{R}\right).$ We chose to fix $M_{S}=M_{A}=M_{P}=M$ so that an ’order *M*’ applied to the whole model. Models of different orders are characterized by how much information on plastic responses is included: order 0 does not estimate any plasticity, order 1 calculates slopes, order 2 additionally fits parabolas, and so on.

This model was fitted for both traits RW and RMD separately (univariate models), and also for RW and RMD simultaneously (multivariate model). In this latter case, the effects *s*, *a* and *p* were estimated for each trait; the variance-covariance matrices Σ gathered the variances of each combination of trait and order and the covariances between them all; the residual variance $\sigma_{R}^{2}$ was independent for each trait.

The model was fitted by Markov chain Monte Carlo (MCMC) with the same priors as in Marchal *et al.* (2017) (Hadfield 2010; R Core Team 2017): parameter expansion was used on the genetic variance-covariance matrices, and flat improper priors were set on the permanent environment variance-covariance matrices as well as on the residual variances. All chains were $5.5\times 10^{6}$ iterations long, with $5\times 10^{5}$ iterations burn-in and a thinning of $5\times 10^{3}$. Point estimations from chains were maximum *a posteriori*, and 95% credible intervals (CIs) were computed where appropriate. The quality of fitting depending on the order was assessed using coefficients of determination $R^{2}$.

### Estimation of genetic parameters

The additive variances ($\sigma_{A:\text{RW}}^{2}\left(x\right)$ and $\sigma_{A:\text{RMD}}^{2}\left(x\right)$) and covariance between RW and RMD ($c_{A}\left(x\right)$) were calculated from the multivariate random regression model as functions of the environmental gradient *x*. The additive variance-covariance matrix decomposes into $\Sigma_{A}=\left[\begin{array}{cc} V_{A:RW} & C_{A}\\ C_{A}^{\text{t}} & V_{A:RMD} \end{array}\right]$ where $V_{A:RW}$ and $V_{A:RMD}$ are the variance-covariance sub-matrices for each trait, $C_{A}$ is the across-traits covariance sub-matrix, and the operator: ${}^{\text{t}}$ indicates a transposition. The additive variance of a trait *T* is the variance of the additive performances within the population for this trait, that is the variance of a linear combination:

$$\sigma_{A:T}^{2}\left(x\right)=\text{var}\left(\sum_{m=0}^{M_{A}}a_{jm:T}L_{m}\left(x^{\prime }\right)\right)=L\left(x^{\prime }\right)V_{A:T}L^{\text{t}}\left(x^{\prime }\right)$$

Where $L\left(x^{\prime }\right)=\left[L_{0}\left(x^{\prime }\right)L_{1}\left(x^{\prime }\right)\ldots L_{M_{A}}\left(x^{\prime }\right)\right]$ The permanent environment variance $\sigma_{P:T}^{2}\left(x\right)$ was computed in the same way. Similarly, the additive covariance between the 2 traits was computed as:

$$c_{A}\left(x\right)=L\left(x^{\prime }\right)C_{A}L^{\text{t}}\left(x^{\prime }\right)$$

Finally, the permanent environment covariance $c_{P}\left(x\right)$ was computed in a similar way. Narrow-sense heritabilities and additive correlation were then computed respectively:

$$h_{T}^{2}\left(x\right)=\frac{\sigma_{A:T}^{2}\left(x\right)}{\sigma_{A:T}^{2}\left(x\right)+\sigma_{P:T}^{2}\left(x\right)+\sigma_{R:T}^{2}}$$

and:

$$r_{A}\left(x\right)=\frac{c_{A}\left(x\right)}{\sqrt{\sigma_{A:\text{RW}}^{2}\left(x\right)\sigma_{A:\text{RMD}}^{2}\left(x\right)}}$$

For hybrids, narrow-sense heritabilities and additive correlations were computed for each of the parental contributions $g_{E}$ and $g_{J}.$ These heritabilities $h_{HE}^{2}$ and $h_{HJ}^{2}$ quantify the proportion of hybrid phenotypic variance that is due to the additive inheritance from each parental side (Marchal *et al.* 2017). In the same way, the additive correlations $r_{HE}$ and $r_{HJ}$ are the correlations between the additive contributions for the 2 traits from each parental side. Therefore, on the EL side and given that $\Sigma_{HE}=\left[\begin{array}{cc} V_{HE:RW} & C_{HE}\\ C_{HE}^{\text{t}} & V_{HE:RMD} \end{array}\right]$, for a trait *T*:

$$\sigma_{HE:T}^{2}\left(x\right)=L\left(x^{\prime }\right)V_{HE:T}L^{\text{t}}\left(x^{\prime }\right)$$

from which:

$$h_{HE:T}^{2}\left(x\right)=\frac{\sigma_{HE:T}^{2}\left(x\right)}{\frac{1}{2}\sigma_{HE:T}^{2}\left(x\right)+\frac{1}{2}\sigma_{HJ:T}^{2}\left(x\right)+\sigma_{P:T}^{2}\left(x\right)+\sigma_{R:T}^{2}}$$

and for the covariance parameters between the 2 traits:

$$c_{HE}\left(x\right)=L\left(x^{\prime }\right)C_{HE}L^{\text{t}}\left(x^{\prime }\right)$$

from which:

$$r_{HE}\left(x\right)=\frac{c_{HE}\left(x\right)}{\sqrt{\sigma_{HE:\text{RW}}^{2}\left(x\right)\sigma_{HE:\text{RMD}}^{2}\left(x\right)}}$$

and idem on the JL side.

### Simulation: evaluation of the random regression model with single record per individual

We used the software Metagene to simulate data. The functioning of this simulator to derive *in silico* populations with phenotypic plasticity records is detailed in Supplementary 3. Basically, the genotypic effect at a given locus was set as a function of the environment $\alpha \left(x\right)=\alpha_{0}+\alpha_{1}\left(x+\delta \right)+\alpha_{2}{\left(x+\delta \right)}^{2},$ where parameters $\alpha_{0},$ $\alpha_{1},$ $\alpha_{2}$ and *δ* defined the parabola that was associated to each genotype in a set of *X* diallelic loci constituting the genome. Then, for each independent simulation, the simulator randomly sampled a genome for each founder, produced the mating between founders, the new offspring genomes, and returned their phenotypic plasticity in the form of longitudinal records over *Y* environments. We parameterized the allelic effects in such a way that the additive reaction norms were very interactive, that is, the ranking of the parents varied along the environmental gradient due to slopes and parabolas.

The simulated mating design consisted of a full diallel between 10 monoecious founders, excluding selfs. Unlike the real mating design previously described, the simulated mating design involved a single species so there was a single additive variance to estimate. Each combination of parents (A×B) produced n/2 sibs, so that the size of a full-sib family (A×B + B×A) was *n*. The environmental gradient was divided in *n* random positions, each position being defined by an environmental value *x*. Each of these environments hosted one sib per family, and as many individuals as families. The progenies’ phenotypes, their pedigree, and the environmental values *x* were included in the analysis. As there were no repeated observations of the individuals, the permanent environment was unidentifiable, it was neither simulated neither included in the analysis.

We tested 4 different scenarios, resulting from the combination of low (0.1) and high (0.6) heritabilities with small (20) and large (120) family sizes. Thus, these scenarios measured the importance of the quantity (*i.e.*, the number of progenies) and quality (*i.e.*, the heritability) of information for genetic inference. For each scenario, 100 independent simulations were run and analyzed. The analysis was performed using pure species univariate random regressions:

$$y_{i}\left(x\right)=\sum_{m=0}^{M}\mu_{m}L_{m}\left(x^{\prime }\right)+\sum_{m=0}^{M}a_{im}L_{m}\left(x^{\prime }\right)+r_{i}$$

where $y_{i}\left(x\right)$ was the only observation for individual *i*, from environment *x*, *μ* were the parameters for the mean reaction norm, *a* were the additive effects such as $a∼N\left(0,\Sigma_{A}⊗A\right)\text{,}$ and *r* was the unstructured residual.

The chains were $1.5\times 10^{5}$ iterations long, with $5\times 10^{4}$ iterations burn-in and a thinning of $10^{3}.$ We measured the ability of the random regression to infer additive components of the parental reaction norms. To do so, we defined the accuracy of the model as the correlation between the parents’ predicted additive performances and their true simulated additive performances (Mrode and Thompson 2005), and we computed it at each point of the environmental gradient. The reaction norms of the parents were only inferred for the ranges on which the progenies were tested.

### Prediction of the larch reaction norms with single records per individuals

Finally, we also investigated the possibility to infer reaction norms from single records per individuals using the empirical larch data. We randomly sampled one observation per tree in the dataset, and used it to fit the random regression model. The model we used was the same as for the complete empirical larch dataset, except without the permanent environment component that became unidentifiable.

### Data availability

Our datasets are available at the INRA repository GnpIS https://data.inra.fr/dataset.xhtml?persistentId=doi:10.15454/REMJWZ&version=DRAFT (https://doi.org/10.15454/REMJWZ).

The Metagene simulator is available on the NOVELTREE project page https://data.inra.fr/dataset.xhtml?persistentId=doi:10.15454/ELCSZI (https://doi.org/10.15454/ELCSZI). Supplemental material available at Figshare: https://doi.org/10.25387/g3.7211597.

## Results

### Quality of model fitting to real data

**Figure 2:**
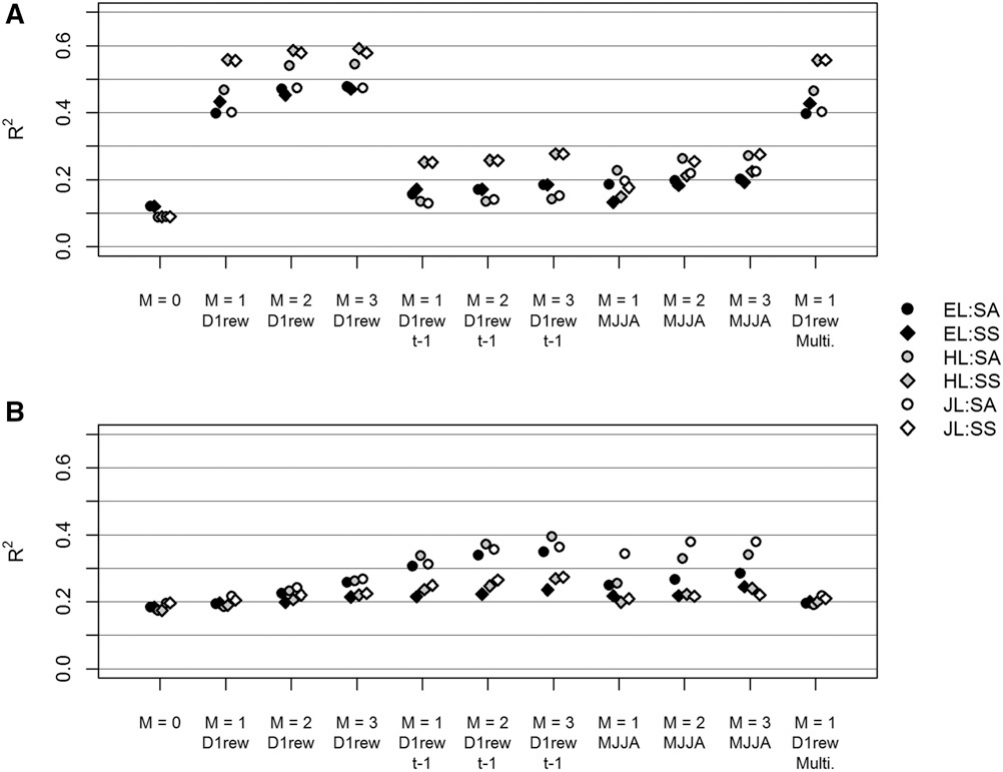
Coefficients of determination ($R^{2}$) for ring width (RW) (a) and ring mean density (RMD) (b), depending on the model specification. The polynomial order (*M*) varied from 0 to 3. Three covariates were compared: the first decile of the soil daily relative extractable water (D1rew) for the current year (year *t*, implicit) and for the previous year (year t−1, specified), and the sum of daily differences between precipitation and potential evapotranspiration from May to July (MJJA) for the current year. The last model specification (’Multi.’) corresponds to 1${}^{\text{st}}$ order regression with the covariate D1rew of the current year *t*, but in this case the model was multivariate and RW and RMD were analyzed simultaneously.

The order 0 model (fixed and random intercepts) explained between 8.9% and 12.1% of RW variance depending on taxa and site combination (Figure 2). The addition of fixed and random slopes (1^st^ order) along D1rew of the current year (*t*) greatly improved the model, allowing it to account for 39.9% (EL in SA) to 55.8% (HL in SS) of the variance. Analyzing higher orders (order 2 and order 3 along D1rew of the current year) slightly increased the $R^{2}$ (by 5.1% on average compared to order 1). Such high $R^{2}$ were not reached using D1rew of the previous year, nor using the simpler index MJJA. For RMD, on the contrary, order 0 or higher orders led to very close $R^{2}$ (Figure 2). Using D1rew of the previous year instead of that of the current year led to a marginal improvement of $R^{2}$ that happened only in SA, reaching up to 39.4% for HL with order 3.

**Figure 3:**
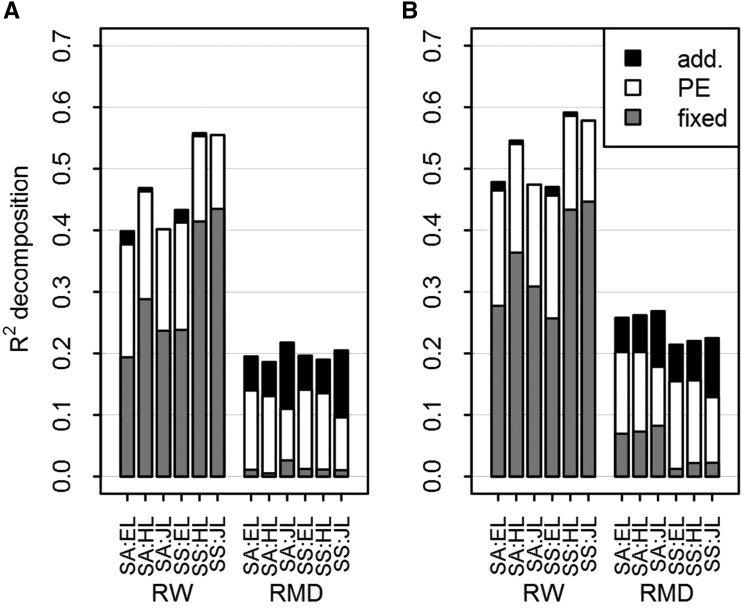
Decomposition of the coefficients of determination ($R^{2}$) obtained with the order 1 model (a) and with the order 3 model (b), for each trait: ring width (RW) and ring mean density (RMD), and for each combination of site (SA or SS) and taxon (EL, HL or JL). The environmental gradient was the first decile of the daily relative extractable water for the current year. The proportion of variance explained by each component is presented: genetic additive effects (in black), permanent environment (in white), and fixed terms (in gray).

**Figure 4:**
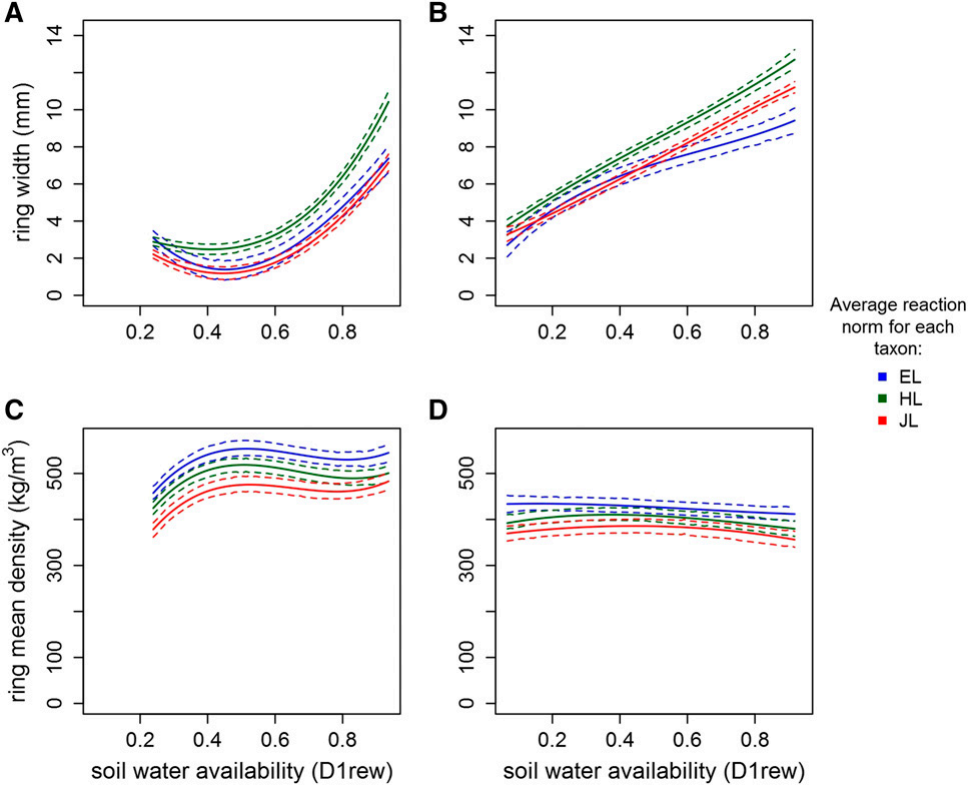
Reaction norms of ring width (a, b) and ring mean density (c, d) along the first decile of the daily relative extractable water (D1rew), in the sites SA (a, c) and SS (b, d), for each taxon: European larch (in blue, EL), Japanese larch (in red, JL) and their hybrid (in green, HL). These average reaction norms at the taxon level represent the fixed components of the order 3 random regressions. Dashed lines: 95% credible intervals.

Heterosis expressed mostly in radial growth, that was better explained by D1rew of the current year. For this reason, we focused on this environmental gradient only, and all the subsequent results refer to random regression models along D1rew at year *t*. The gain in $R^{2}$ with orders beyond 1 was overall due to more variance explained by the fixed component of the models, as shown in Figure 3. Therefore, we present in Figure 4 and Fig. S10 (Supplementary 4) the taxon scale reaction norms (*i.e.*, the fixed component) estimated from the order 3 univariate random regression models. Then, for parsimony consideration and because increasing the order did not increase the portion of $R^{2}$ captured by the random effects (Figure 3), the multivariate random regression models (RW and RMD analyzed simultaneously), from which the genetic variance and covariance parameters were estimated, were fitted with order 1. The $R^{2}$s of the multivariate models were also presented in Figure 2 and were very similar to their univariate counterparts.

### Stability of the heterosis and its dependence on the water availability

The ranking in RW performance between EL and JL varied depending on the site: EL performed better in SA while JL did better in SS (Figure 4). Meanwhile, the superiority of the hybrid over both its parental references, hence best-parent heterosis, occurred in the two sites and over the whole range of D1rew.

For any taxon and in any site, RW was plastic as it increased with increasing water availability (Figure 4). The three taxa showed however different curves along the D1rew, being close to each other when water availability was minimal (D1rew close to 0), and splitting apart with increasing water availability, where the superiority of HL over its parental references was the highest. Thus, HL had the steepest reaction norm in any site, as shown in Table 1. The gain in superiority for RW of HL over its parental references due to increasing D1rew ranged between +3.26 mm (HL *vs.* EL in SA) and +1.04 mm (HL *vs.* JL in SS). For high D1rew, the CIs of the HL reaction norm were not overlapping with those of the parental species.

**Table 1:** Estimation of the average slope for each taxon (1^st^ order parameter of the fixed component of the order 3 model) for ring width along the water availability gradient (D1rew), in each site (SA and SS). Point estimate: maximum *a posteriori* and in brackets: 95% CI

	Site SA	Site SS
European larch	0.4 (0.1 - 0.8)	2.5 (2.2 - 2.8)
Japanese larch	1 (0.7 - 1.3)	3.4 (3.2 - 3.5)
Hybrid	2.2 (1.9 - 2.4)	3.6 (3.4 - 3.9)

On the opposite, the trait RMD showed neither heterosis nor plasticity. The hybrid ranged between both its parents, and all the norms of reaction were almost flat for this trait, showing no conspicuous pattern of variation along the water availability gradient D1rew.

### Heritabilities and genetic performances along the water availability gradient

**Figure 5:**
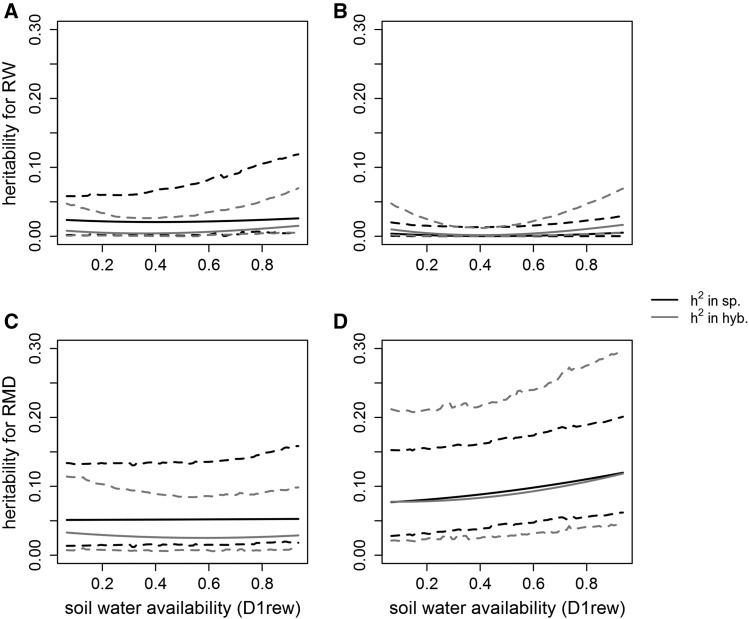
Narrow-sense heritabilities along the first decile of the daily relative extractable water (D1rew), for European larch (a, c) and Japanese larch (b, d), for the traits: ring width (a, b) and ring mean density (c, d). These heritabilities were computed from the variance parameters estimated with the multivariate, order 1 random regressions. Black line: narrow-sense heritability in pure species crosses; and gray line: narrow-sense heritability in hybridization (*i.e.*, proportion of hybrid phenotypic variance that is due to the additive inheritance from each parental side). Dashed lines: 95% credible intervals.

All the narrow-sense heritabilities we estimated were very low (Figure 5). Low heritabilities were overall due to high residual variances in comparison to the lower additive and permanent environment variances (Figure 3). Despite this residual noise, some extreme parental performances of contrasting genotypes were different for both traits with statistical credibility (Supplementary 4, Fig. S6 - S7).

Ring width heritabilities were close to 0 (Figure 5). The signal for performance contrasts for RW in pure species was also very weak, but both species showed contrasted performances in hybridization as the water availability increased; some of these contrasts were supported by non-overlapping 95% CIs when D1rew was high (Supplementary 4, Fig. S7).

Heritabilities for RMD were higher than those of RW, especially on the JL side for which they varied between 0.08 and 0.12 both in pure species and in hybridization (Figure 5). Pure species heritability and heritability in hybridization were very close for JL, and they both increased with water availability, though the increase was not supported by statistical confidence (the CIs were large and encompassed the whole variation). Increasing contrasts between individual performances, supported by some non-overlapping 95% CIs for high D1rew (Supplementary 4, Fig S7), may reflect this possible gain in heritability along the D1rew gradient for RMD on the JL side. Noteworthily, the ranking of the 9 JL parents’ performances for RMD was consistent in pure species and in hybridization (Supplementary 4, Fig S6).

### Correlations along the water availability gradient

**Figure 6:**
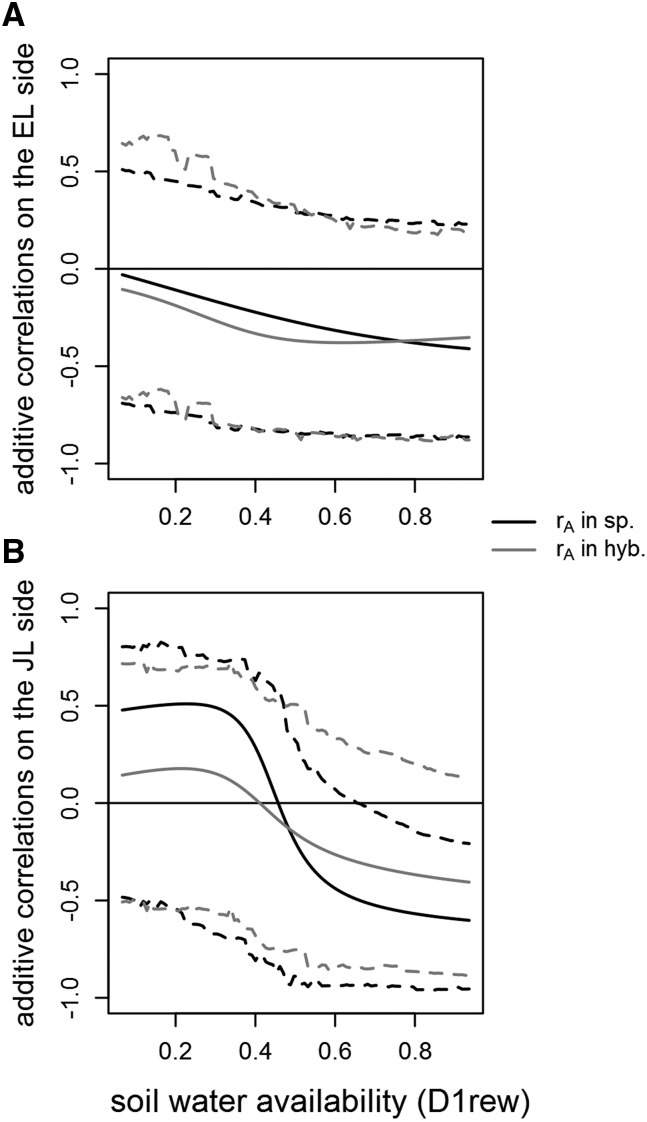
Additive correlations between ring width and ring mean density along the first decile of the daily relative extractable water (D1rew), for European larch (a) and Japanese larch (b). These correlations were computed from the variance and covariance parameters estimated with the multivariate, order 1 random regressions. Black line: correlation in pure species crosses; and gray line: correlation in hybridization (*i.e.*, correlation between the additive contributions for the 2 traits from the corresponding parental side). Dashed lines: 95% credible intervals.

The additive genetic correlation between RMD and RW showed similar patterns between pure species and their respective contributions in hybridization (Figure 6). The additive correlation decreased slightly along the gradient from around 0 to -0.41 in EL pure species (-0.35 in hybridization). On the JL side, it started from a positive correlation of 0.48 in pure species (0.14 in hybridization) and it steeply switched to a negative correlation of -0.60 (-0.41 in hybridization). Uncertainty was high around this parameter, however, for D1rew high enough, the 95% CIs excluded 0 (completely or mostly) on the JL side (in pure species or in hybridization, respectively) (Figure 6). Due to the higher genetic variance for RW for JL in hybridization than in pure species, the negative correlation pattern was especially visible in the JL performances in hybridization: for the highest D1rew, the ranking between RW and RMD was almost inverted (Supplementary 4, Fig S6).

The permanent environment correlation between RW and RMD was negative for HL and JL, and did not vary along the environmental gradient. It was also null to negative for EL (Supplementary 4, Fig S8). This means that the sum of effects that the model did not explicitly account for (*i.e.*, micro-environment, competition between trees, non-additive genetic effects, etc.) tended to induce a negative correlation between radial growth and wood density.

### Simulation: accuracy of the random regression model With single record per individual

**Figure 7:**
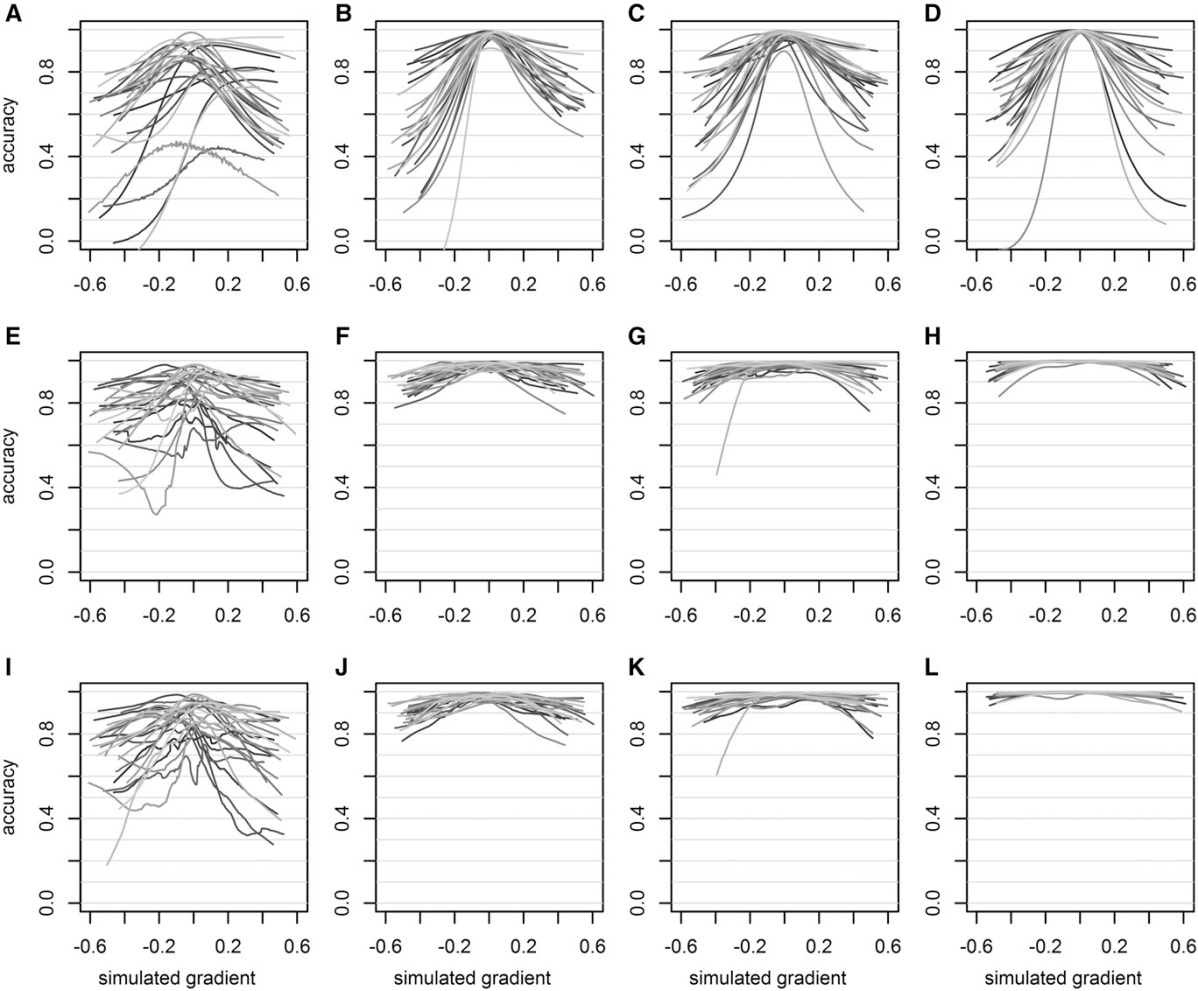
Accuracy of the predictions of parents’ additive reaction norms in each simulated scenario: (1) $h^{2}=0.1$ and n=20 (a, e, i), (2) $h^{2}=0.1$ and n=120 (b, f, j), (3) $h^{2}=0.6$ and n=20 (c, g, k), and (4) $h^{2}=0.6$ and n=120 (d, h, l); for 100 simulations in each scenario, and for each order of random regression: order 0 (a-d), order 1 (e-h) and order 2 (i-l). Each gray curve is the accuracy of 1 simulation.

Using simulated data, we evaluated the ability of the random regression model to predict the additive component of reaction norms from family series of single observations per environment. The accuracy of the model depended on the scenario and on the order of the random regression (Figure 7). The $1^{\text{st}}$ scenario (n=20 progenies, $h^{2}=0.1$) showed very poor predictive abilities, independently of the order. When a larger number of progenies (n=120) or a higher heritability ($h^{2}=0.6$) was available, the accuracy was still low for order 0 (fixed and random intercept model) but it greatly increased with order 1 (addition of fixed and random slopes). However, in each case (n=120 or $h^{2}=0.6$) no further accuracy was gained from order 1 to order 2. Only the accuracy for the last scenario (both n=120 and $h^{2}=0.6$) increased with order 2 (addition of fixed and random parabolas). The accuracy for the last scenario analyzed with order 2 model ranged between 0.905 and 1 depending on the simulation and on the position along the environmental gradient.

The order 0 models were not able to estimate the additive variance properly (Supplementary 4, Fig. S9). From order 1 and over, the ability of the model to estimate the additive variance appeared overall dependent on the heritability. Indeed, the estimated additive variance was close to the true one in scenarios 3 and 4 (both $h^{2}=0.6$) with order 1 or 2. With lower heritability ($h^{2}=0.1$) and order 1 or 2, the additive variance was overestimated.

Finally, we also fitted random regression using only a single observation per tree from the empirical dataset. As show in Supplementary 4, Fig. S10, the average taxon-scale reaction norms thus estimated were close to the ones estimated using the whole dataset (Figure 4). The uncertainty was much higher using a single observation per tree, however, both the slope of the reaction norms and the ranking of the taxa were well preserved.

## Discussion

In this study, we constructed the reaction norms of annual wood-formation traits along a water availability gradient in a larch multi-site diallel mating experiment. The reaction norms were fitted using random regression modeling, allowing to investigate the changes in heritability and genetic correlations along the gradient. Our study was complemented by using simulations involving the same analytical approach, where we evaluated the ability of the random regression model to estimate the additive component in a frequent phenotypic plasticity experimental setting: that of parental reaction norms from non-repeated observations of their progenies as data.

The annual ring width was plastic and as expected increasing water availability allowed a higher radial growth (Figure 4). At the taxon level, the hybrids performed better than their parents in each of the two sites of the study. This alternative modeling confirmed our previous findings (Marchal *et al.* 2017), in that hybrid larch had a stable superiority across sites. However, within each site, HL demonstrated more plasticity than its parental references: indeed, under water stress all taxa produced a similarly narrow ring, whereas in favorable conditions of higher water availability the HL expressed superiority over its parental references. In other words, the HL reaction norms were steeper than those of the parental counterparts over the same environmental gradient.

On the contrary, the second trait under study, RMD, was not plastic for this water availability gradient and expressed no heterosis in any site. Although RMD displayed higher narrow-sense heritabilities than RW, it did not reach high values, with a global maximum of only 0.12. Despite the weak genetic signal, we represented the genetic parameters and the performances of the 18 parents of the diallel as functions of the water availability gradient. Some performance functions were distinct with statistical credibility, only when the water availability index was high enough. Interestingly, on the Japanese larch side, the additive correlation switched from being uncertainly positive to reliably negative as water availability increased (Figure 6). The seemingly emergence of this negative correlation may be explained by a genetically variable increase in the early wood / late wood ratio with increasing water availability, with early (spring) wood being generally less dense than that of late (summer) wood (Figure 1). However, we need to look more carefully to other ring traits (as did, *e.g.*, Bryukhanova and Fonti 2013) and their respective correlations before proposing any causal explanation, with the goal to better understand the structure of the genetic variability of larch wood plasticity.

We obtained fairly high $R^{2}$ for the reaction norms models, suggesting that water plays an important role in the tree ring phenotypic plasticity. We evaluated a simpler environmental factor (sum of daily rain minus potential evapotranspiration from May to August) but it showed a lower $R^{2},$ highlighting the relevance of our water availability index ’D1rew’. It should be noted, however, that our water balance model has not been field-calibrated, and it should then be considered with care if generalizations are to be made. Moreover, the index D1rew gives no indication on the distribution of the driest days along the year. The timing of a water deficit, in spring or in summer, could have more or less effect on different ring traits; for instance, those relative to early or late wood, or to the transition between the two. It also has to be said that the relation between water balance and radial growth that we showed in the present study does not necessarily imply a direct causality. Indeed, other factors may play a role in the observed plastic response. For instance, heat affects directly the photosynthetic efficiency and the resources that may be allocated to growth (Rennenberg *et al.* 2006). Heat and drought being highly correlated, their effects could well be confounded to some extent. More broadly, the environment has a multivariate nature. Soil, climate, but also competition with neighboring trees are known to affect the tree’s growth. We isolated what we expected to be one of the most important environmental factor for radial growth, yet the existence of an important site effect pinpoints the fact that some other environmental factors might be involved in the tree ring phenotypic plasticity. Identifying relevant environmental factors of plant plastic reactions is an open area of research, notably in the context of global warming. The present study did not aim explicitly at the identification of relevant environmental triggers, rather it presented an approach that could help in such identification.

We studied phenotypic plasticity at two levels. The first level was spatial, at the across-site scale, and the second level was longitudinal, at the individual scale. At the across-site scale, heterosis was shown to be stable, supporting the common statement that hybrids are more stable across macro-environmental sites than their parental counterparts (Gallais 2009). Specifically, the ranking of the parents species varied across sites whereas hybrid was invariably the highest performing taxon, in what could be qualified as hybrid homeostasis according to the theory developed by Knight (1973). The spatial plasticity is generally, and historically, the one that interests breeders the most because of the operational implications for the deployment of varieties. Using non-linear random regressions, Marcatti *et al.* (2017) fitted eucalyptus growth reaction norms along a gradient of spatial environments. The spatially distributed climatic environment was described with principal component analysis to account for its multivariate nature. This method is very appealing in order to deal with spatial phenotypic plasticity in tree breeding. However, Marcatti *et al.* (2017)’s approach could be further improved by accounting for genetic information, such as the one from a pedigree and from which quantitative genetic parameters could be estimated (Hinkelmann 1974; Falconer and Mackay 1996). In this context, the use of random regressions as defined by Kirkpatrick and Heckman (1989) and presented in this paper, or other covariance functions, would be relevant.

The second level of phenotypic plasticity that we addressed was longitudinal, along the year-to-year water availability gradient. The resulting reaction norms were strongly influenced by the sites. Indeed, our study steps in the direction that tree ring longitudinal phenotypic plasticity should be seen as a plastic trait in itself, varying spatially (De Luis *et al.* 2013; Natalini *et al.* 2016), varying with long-term trends such as global warming (Natalini *et al.* 2016), and varying with the level of competition between neighboring trees in wet years (Carnwath and Nelson 2016). Growth recovery, the ability for trees to produce large rings the years following a drought event, is also site-dependent (Gazol *et al.* 2017). Besides the site effect, a substantial part of the individual variation was shown to occur between taxa. Unlike what we initially expected, hybrid larch reaction norms for radial growth along the water availability gradient were not flatter than the ones from its parental species: on the contrary, they displayed more plasticity and were steeper than the parental ones. Interestingly, this increase in longitudinal plasticity for HL incrementally contributes to the construction of heterosis that was observed on an integrative scale across years, namely in the total circumference (Marchal *et al.* 2017).

The molecular and physiological mechanisms behind the increased plasticity of hybrid larch remain open questions. Longitudinal plasticity as shown here is still a novel approach, with less immediate application to current plant breeding, unlike spatial plasticity. It certainly opens up new possibilities whenever long-time series are available, where extreme events, such as the 2003 drought in France (Bréda *et al.* 2006; Rennenberg *et al.* 2006), are recorded. Some initiatives, for instance, compared dead trees *vs.* alive neighbors immediately after extreme climatic events for their past wood records (Martinez-Meier *et al.* 2008), finding that both classes had long-term distinctive patterns of reaction. This kind of study could well be undertaken with a random regression approach to gain insight in the quantitative genetics of such longitudinal patterns and their environmental drivers, and be of potential use ultimately for breeders.

One eventual problem with longitudinal data are autocorrelation. In order to minimize its effects, several authors propose an extra step that consists in fitting an autocorrelation model on the tree ring series. The resulting residuals, that are more independent than the raw data, are then used as the response variable in the subsequent phenotypic plasticity models (Bryukhanova and Fonti 2013; De Luis *et al.* 2013). In this study, we did not do so because the chronology was somehow already involved in the environmental variable. Indeed, as the trees grew older, the stand LAI increased, and so did the transpiration, making water generally less available. Though this trend was not so strong (rain and potential evapotranspiration during the growing season were the main drivers of D1rew, as seen on Supplementary 1, Fig. S2), we did not want to account twice for the same chronological effect, and therefore we chose to work with raw data instead. The much weaker explicative power of D1rew of the previous year compared to that of D1rew of the current year supports our decision. We acknowledge though that there remains a risk of non-controlled autocorrelation in the data, in particular due to the trees’ ontogeny and to the onset of competition between trees (Sánchez *et al.* 2013).

The simulations showed that it was possible to estimate the additive component of reaction norms using only singular observations of related individuals. However, the quantity and the quality of information (*i.e.*, respectively, the number of related individuals and the heritability) were key factors to estimate properly the additive components of reaction norms. Although the simulation was not meant to mimic the real case in the genomic layout of effects, it pinpointed the eventuality of potential biases in the estimation of the genetic variances (Supplementary 4, Fig. S9). As emphasized by Misztal *et al.* (2000), a limitation of the model used in the present study is the lack of covariance function for the residuals. This limitation could be a source of bias in the estimation of variance components and of heritabilities. Unfortunately, this feature was not available yet with the software we used. Indeed, we fitted linear mixed models in which the covariance functions were implicit and computed from the covariance between the regression coefficients. On the other hand, despite this issue with the variance, the simulated additive reaction norms could be properly estimated in most scenarios. Moreover, we also showed that the average reaction norm at the taxon level could be estimated from a single observation per genotype using the empirical larch data.

Random regression is already used for the modeling of reaction norms, especially in dairy cattle for which industry produces a large flow of longitudinal data (*e.g.*, Kolmodin *et al.* 2002; Windig *et al.* 2006; Santana *et al.* 2017). However, plasticity has been suggested (Bradshaw 1965) and demonstrated (Murren *et al.* 2014) to be of special importance in plants. Indeed, because they are sessile, plants have to face their environment in a different way than animals that are capable of behavioral responses and locomotion. Manifestations of phenotypic plasticity have been reported in several perennial crops. For instance, grape vine manifests phenotypic plasticity in terms of fruit weight and chemical composition (Dai *et al.* 2011). Even in equatorial regions, oil palm is able to react to subtle variations in photoperiod and drought events by changing its bunch productivity (Legros *et al.* 2009). Cherry tree phenology reacts promptly to climate, notably heat, with global warming expected to bring flowering a month forward (Allen *et al.* 2014). Ismaili *et al.* (2016) showed a significant genotype-by-year interaction in apricot tree, and recommend the use of mixed models for the analysis of perennial plants’ longitudinal data. All these examples could be good candidates for analysis based on covariance functions. Indeed, the possibility to define quantitative genetic parameters as functions of the environment and to model the additive contributions to phenotypic plasticity opens wide perspectives in terms of selection (De Jong and Bijma 2002), especially in a global warming context (Koski 1996). In that sense, Chevin and Hoffmann (2017) pinpoint the importance of inferring the complex functions underlying plastic responses, notably those modeled under extreme environments, as essential steps to address evolutionary questions related to the amount of genetic differences in plasticity to extreme events, and to what extent these reflect heritability. The same authors also argue that comprehending the evolutionary dynamic of phenotypic plasticity would require a fine knowledge on the genetic constraints that could operate across environments. Our study contributed to illustrate methodologically several of the perspectives highlighted by Chevin and Hoffmann (2017).

Like trees, other organisms naturally accumulate growth records that reflect their reaction to past environmental conditions, etched in hard organs that grow incrementally: for instance, fish otoliths, mollusc shells, corals, whale ear plugs, ibex horns, *etc*. (reviewed by Morrongiello and Thresher 2015). Morrongiello and Thresher (2015) advocate for the use of random regression for the analysis of such natural records of longitudinal data, especially with regards to these species’ phenotypic plasticity. On a prospective review concerning new integrative ways to assess phenotypic plasticity, Stinchcombe *et al.* (2012) already highlighted the potential advantages of using random regression in the context of non-linear continuous reaction norms, notably to study growth curves matched by chronological age.

The random regression framework as developed by Kirkpatrick and Heckman (1989) exhibits two particular strengths that deserve to be emphasized once more. First, the use of orthogonal base functions, such as Legendre polynomials, allows the fit of virtually any shape of growth curves or reaction norms. Although the example we presented here did not illustrate this need, neglecting curvature when studying evolutionary divergence in reaction norms leads to a risk of missing critically important information (Murren *et al.* 2014). The traditional use of *x* and $x^{2}$ as covariates should be avoided in any case, given the high correlation that binds the identity and the square (and any power) functions.

A second point of interest is the fact that genetic information can be taken into consideration in the model, which can be of relevance not only for breeding but also in ecology studies looking for drivers and patterns of natural selection (Brommer *et al.* 2005). As we illustrated with the results of our simulations, this possibility opens up the use of a random regression framework to species whose individuals do not cumulate in any known form longitudinal records of their plastic responses. In this sense, our simulation provided an example of random regression being an alternative to traditional methods: related individuals can indeed give access to the additive component of the reaction norms, and this can likely be extrapolated to isofemale lines (Gibert *et al.* 2004) or half-sib families (Valladares *et al.* 2006). Finally, the pedigree information can be conveniently replaced by molecular information (*e.g.*, Ly *et al.* 2018), extending the potential of the random regression framework beyond the limitation of our capability to realize time-consuming, sometimes impossible, artificial mating. This also opens up the possibility to realize genome-wide association studies in order to detect the genetic variants involved in phenotypic plasticity.

## References

[bib1] Allen J. M. Terres M. A. Katsuki T. Iwamoto K. Kobori H., 2014 Modeling daily flowering probabilities: expected impact of climate change on Japanese cherry phenology. Glob. Change Biol. 20: 1251–1263. 10.1111/gcb.12364 23966290

[bib2] Apiolaza L. A. Garrick D. J., 2001 Analysis of longitudinal data from progeny tests: some multivariate approaches. For. Sci. 47: 129–140.

[bib3] Bradshaw A. D., 1965 Evolutionary significance of phenotypic plasticity in plants. Adv. Genet. 13: 115–155.

[bib4] Bréda N. Huc R. Granier A. Dreyer E., 2006 Temperate forest trees and stands under severe drought: a review of ecophysiological responses, adaptation processes and long-term consequences. Ann. For. Sci. 63: 625–644. 10.1051/forest:2006042

[bib5] Brommer J. E. Merilä J. Sheldon B. C. Gustafsson L., 2005 Natural selection and genetic variation for reproductive reaction norms in a wild bird population. Evolution 59: 1362–1371. 10.1111/j.0014-3820.2005.tb01785.x 16050111

[bib7] Bryukhanova M. Fonti P., 2013 Xylem plasticity allows rapid hydraulic adjustment to annual climatic variability. Trees (Berl.) 27: 485–496. 10.1007/s00468-012-0802-8

[bib8] Carnwath G. C. Nelson C. R., 2016 The effect of competition on responses to drought and interannual climate variability of a dominant conifer tree of western North America. J. Ecol. 104: 1421–1431. 10.1111/1365-2745.12604

[bib9] Chevin L.-M. Hoffmann A. A., 2017 Evolution of phenotypic plasticity in extreme environments. Philos. Trans. R. Soc. Lond. B Biol. Sci. 372: 20160138 10.1098/rstb.2016.0138 28483868 PMC5434089

[bib10] Dai Z. W. Ollat N. Gomès E. Decroocq S. Tandonnet J.-P., 2011 Ecophysiological, genetic, and molecular causes of variation in grape berry weight and composition: a review. Am. J. Enol. Vitic. 62: 413–425. 10.5344/ajev.2011.10116

[bib11] De Jong G. Bijma P., 2002 Selection and phenotypic plasticity in evolutionary biology and animal breeding. Livest. Prod. Sci. 78: 195–214. 10.1016/S0301-6226(02)00096-9

[bib12] De Luis M. Čufar K. Di Filippo A. Novak K. Papadopoulos A., 2013 Plasticity in dendroclimatic response across the distribution range of Aleppo pine (*Pinus halepensis*). PLoS One 8: e83550 10.1371/journal.pone.0083550 24391786 PMC3877073

[bib13] Falconer D. S. Mackay T. F., 1996 Introduction to quantitative genetics, Ed. 4th Longman, Harlow.

[bib14] Fallour-Rubio D. Guibal F. Klein E. K. Bariteau M. Lefèvre F., 2009 Rapid changes in plasticity across generations within an expanding cedar forest. J. Evol. Biol. 22: 553–563. 10.1111/j.1420-9101.2008.01662.x 19170817

[bib15] Gallais A., 2009 Hétérosis et variétés hybrides en amélioration des plantes, Quae, Versailles.

[bib16] Gazol A. Camarero J. J. Anderegg W. R. L. Vicente-Serrano S. M., 2017 Impacts of droughts on the growth resilience of Northern Hemisphere forests. Glob. Ecol. Biogeogr. 26: 166–176. 10.1111/geb.12526

[bib17] Gibert P. Capy P. Imasheva A. Moreteau B. Morin J. P., 2004 Comparative analysis of morphological traits among *Drosophila melanogaster* and *D. simulans*: genetic variability, clines and phenotypic plasticity. Genetica 120: 165–179. 10.1023/B:GENE.0000017639.62427.8b 15088656

[bib18] Granier A. Bréda N. Biron P. Villette S., 1999 A lumped water balance model to evaluate duration and intensity of drought constraints in forest stands. Ecol. Modell. 116: 269–283. 10.1016/S0304-3800(98)00205-1

[bib19] Hadfield J. D., 2010 MCMC methods for multi-response generalized linear mixed models: the MCMCglmm R package. J. Stat. Softw. 33: 1–22. 10.18637/jss.v033.i0220808728

[bib21] Hinkelmann K., 1974 Two-level diallel cross experiments. Silvae Genet. 23: 18–22.

[bib22] Ismaili A. Karami F. Akbarpour O. Nejad A. R., 2016 Estimation of genotypic correlation and heritability of apricot traits, using restricted maximum likelihood in repeated measures data. Can. J. Plant Sci. 96: 439–447. 10.1139/cjps-2015-0253

[bib23] Jamrozik J. Bohmanova J. Schaeffer L. R., 2010 Relationships between milk yield and somatic cell score in Canadian Holsteins from simultaneous and recursive random regression models. J. Dairy Sci. 93: 1216–1233. 10.3168/jds.2009-2585 20172242

[bib24] Janick J., 1999 Exploitation of heterosis: uniformity and stability, pp. 319–333 in The genetics and exploitation of heterosis in crops, American Society of Agronomy-Crop Science Society of America, Madison.

[bib25] Johnson P. C. D., 2014 Extension of Nakagawa & Schielzeth’s *R^2^*_GLMM_ to random slopes models. Methods Ecol. Evol. 5: 944–946. 10.1111/2041-210X.12225 25810896 PMC4368045

[bib27] Kirkpatrick M. Heckman N., 1989 A quantitative genetic model for growth, shape, reaction norms, and other infinite-dimensional characters. J. Math. Biol. 27: 429–450. 10.1007/BF00290638 2769086

[bib28] Kirkpatrick M. Lofsvold D. Bulmer M., 1990 Analysis of the inheritance, selection and evolution of growth trajectories. Genetics 124: 979–993. 2323560 10.1093/genetics/124.4.979 PMC1203988

[bib29] Knight R., 1973 The relation between hybrid vigour and genotype-environment interactions. Theor. Appl. Genet. 43: 311–318. 10.1007/BF00275258 24425231

[bib30] Kolmodin R. Strandberg E. Madsen P. Jensen J. Jorjani H., 2002 Genotype by environment interaction in Nordic dairy cattle studied using reaction norms. Acta Agriculturae Scandinavica, Section A. Anim. Sci. 52: 11–24.

[bib31] Koski V., 1996 Breeding plans in case of global warming. Euphytica 92: 235–239. 10.1007/BF00022850

[bib32] Legros S. Mialet-Serra I. Caliman J.-P. Siregar F. A. Clément-Vidal A., 2009 Phenology and growth adjustments of oil palm (*Elaeis guineensis*) to photoperiod and climate variability. Ann. Bot. (Lond.) 104: 1171–1182. 10.1093/aob/mcp214 PMC2766204 19748909

[bib33] Li Y. Suontama M. Burdon R. D. Dungey H. S., 2017 Genotype by environment interactions in forest tree breeding: review of methodology and perspectives on research and application. Tree Genet. Genomes 13: 1–18.

[bib34] Ly D. Huet S. Gauffreteau A. Rincent R. Touzy G., 2018 Whole-genome prediction of reaction norms to environmental stress in bread wheat (Triticum aestivum L.) by genomic random regression. Field Crops Res. 216: 32–41. 10.1016/j.fcr.2017.08.020

[bib35] Marcatti G. E. Resende R. T. Resende M. D. V. Ribeiro C. A. A. S. Dos Santos A. R., 2017 GIS-based approach applied to optimizing recommendations of *Eucalyptus* genotypes. For. Ecol. Manage. 392: 144–153. 10.1016/j.foreco.2017.03.006

[bib36] Marchal A. Muñoz F. Millier F. Sánchez L. Pâques L. E., 2017 Hybrid larch heterosis: for which traits and under which genetic control? Tree Genet. Genomes 13: 92.

[bib37] Martinez-Meier A. Sanchez L. Dalla-Salda G. Gallo L. Pastorino M., 2009 Ring density record of phenotypic plasticity and adaptation to drought in Douglas-fir. For. Ecol. Manage. 258: 860–867. 10.1016/j.foreco.2009.03.021

[bib38] Martinez-Meier A. Sanchez L. Pastorino M. Gallo L. Rozenberg P., 2008 What is hot in tree rings? The wood density of surviving Douglas-firs to the 2003 drought and heat wave. For. Ecol. Manage. 256: 837–843. 10.1016/j.foreco.2008.05.041

[bib39] Meyer K., 1998 Modeling ‘repeated’ records: covariance functions and random regression models to analyse animal breeding data. In *Proceedings of the 6th World Congress on Genetics Applied to Livestock Production*, volume 25, pp. 517–520.

[bib40] Miglior F. Sewalem A. Jamrozik J. Bohmanova J. Lefebvre D. M., 2007 Genetic analysis of milk urea nitrogen and lactose and their relationships with other production traits in Canadian Holstein cattle. J. Dairy Sci. 90: 2468–2479. 10.3168/jds.2006-487 17430951

[bib41] Misztal I. Strabel T. Jamrozik J. Mäntysaari E. A. Meuwissen T. H. E., 2000 Strategies for estimating the parameters needed for different test-day models. J. Dairy Sci. 83: 1125–1134. 10.3168/jds.S0022-0302(00)74978-2 10821589

[bib42] Morrissey M. B. Liefting M., 2016 Variation in reaction norms: statistical considerations and biological interpretation. Evolution 70: 1944–1959. 10.1111/evo.13003 27431762

[bib43] Morrongiello J. R. Thresher R. E., 2015 A statistical framework to explore ontogenetic growth variation among individuals and populations: a marine fish example. Ecol. Monogr. 85: 93–115. 10.1890/13-2355.1

[bib44] Mrode R. A. Thompson R., 2005 Linear models for the prediction of animal breeding values, Ed. 2nd CABI Pub, Wallingford, UK; Cambridge, MA 10.1079/9780851990002.0000

[bib45] Muir B. L. Kistemaker G. Jamrozik J. Canavesi F., 2007 Genetic parameters for a multiple-trait multiple-lactation random regression test-day model in Italian Holsteins. J. Dairy Sci. 90: 1564–1574. 10.3168/jds.S0022-0302(07)71642-9 17297130

[bib47] Murren C. J. Maclean H. J. Diamond S. E. Steiner U. K. Heskel M. A., 2014 Evolutionary change in continuous reaction norms. Am. Nat. 183: 453–467. 10.1086/675302 24642491

[bib48] Nakagawa S. Schielzeth H., 2013 A general and simple method for obtaining *R* from generalized linear mixed-effects models. Methods Ecol. Evol. 4: 133–142. 10.1111/j.2041-210x.2012.00261.x

[bib49] Natalini F. Alejano R. Vázquez-Piqué J. Pardos M. Calama R., 2016 Spatiotemporal variability of stone pine (*Pinus pinea* L.) growth response to climate across the Iberian Peninsula. Dendrochronologia 40: 72–84. 10.1016/j.dendro.2016.07.001

[bib51] R Core Team, 2017 R: a language and environment for statistical computing.

[bib69] Regent Instruments Canada Inc., 2008 WinDENDRO for tree-ring analysis.

[bib52] Rennenberg H. Loreto F. Polle A. Brilli F. Fares S., 2006 Physiological responses of forest trees to heat and drought. Plant Biol. 8: 556–571. 10.1055/s-2006-924084 16773557

[bib54] Santana M. L. Bignardi A. B. Stefani G. El Faro L., 2017 Genetic component of sensitivity to heat stress for nonreturn rate of Brazilian Holstein cattle. Theriogenology 98: 101–107. 10.1016/j.theriogenology.2017.04.052 28601146

[bib55] Schaeffer L. R., 2004 Application of random regression models in animal breeding. Livest. Prod. Sci. 86: 35–45. 10.1016/S0301-6226(03)00151-9

[bib56] Schlichting C. D. Pigliucci M., 1998 Phenotypic evolution: a reaction norm perspective, Sinauer Associates, Sunderland.

[bib57] Sánchez L. Rozenberg P. Bastien C., 2013 Shifting from growth to adaptive traits and competition: the prospect of improving tree responses to environmental stresses, pp. 63–76 in Novel Tree Breeding, Vol. 24 Instituto Nacional de Investigación Tecnologia Agraria y Alimentaria, Madrid.

[bib59] Sánchez-Vargas N. M. Sánchez L. Rozenberg P., 2007 Plastic and adaptive response to weather events: a pilot study in a maritime pine tree ring. Can. J. For. Res. 37: 2090–2095. 10.1139/X07-075

[bib61] Stinchcombe J. R. Function-valued Traits Working Group Kirkpatrick M., 2012 Genetics and evolution of function-valued traits: understanding environmentally responsive phenotypes. Trends Ecol. Evol. 27: 637–647. 10.1016/j.tree.2012.07.002 22898151

[bib62] Stuber C. W. Cockerham C. C., 1966 Gene effects and variances in hybrid populations. Genetics 54: 1279–1286. 17248353 10.1093/genetics/54.6.1279 PMC1211293

[bib64] Valladares F. Sanchez-Gomez D. Zavala M. A., 2006 Quantitative estimation of phenotypic plasticity: bridging the gap between the evolutionary concept and its ecological applications. J. Ecol. 94: 1103–1116. 10.1111/j.1365-2745.2006.01176.x

[bib65] Wang C. Andersson B. Waldmann P., 2009 Genetic analysis of longitudinal height data using random regression. Can. J. For. Res. 39: 1939–1948. 10.1139/X09-111

[bib66] Windig J. J. Calus M. P. L. Beerda B. Veerkamp R. F., 2006 Genetic correlations between milk production and health and fertility depending on herd environment. J. Dairy Sci. 89: 1765–1775. 10.3168/jds.S0022-0302(06)72245-7 16606748

